# SPAD imagers for super resolution localization microscopy enable analysis of fast fluorophore blinking

**DOI:** 10.1038/srep44108

**Published:** 2017-03-13

**Authors:** Ivan Michel Antolovic, Samuel Burri, Claudio Bruschini, Ron A. Hoebe, Edoardo Charbon

**Affiliations:** 1 Applied Quantum Architectures, Department of Quantum Engineering, TU Delft, Netherlands; 2 School of Engineering, EPFL, Switzerland; 3 Cellular Imaging - Core Facility, Academic Medical Centre, University of Amsterdam, Netherlands

## Abstract

sCMOS imagers are currently utilized (replacing EMCCD imagers) to increase the acquisition speed in super resolution localization microscopy. Single-photon avalanche diode (SPAD) imagers feature frame rates per bit depth comparable to or higher than sCMOS imagers, while generating microsecond 1-bit-frames without readout noise, thus paving the way to in-depth time-resolved image analysis. High timing resolution can also be exploited to explore fluorescent dye blinking and other photophysical properties, which can be used for dye optimization. We present the methodology for the blinking analysis of fluorescent dyes on experimental data. Furthermore, the recent use of microlenses has enabled a substantial increase of SPAD imager overall sensitivity (12-fold in our case), reaching satisfactory values for sensitivity-critical applications. This has allowed us to record the first super resolution localization microscopy results obtained with a SPAD imager, with a localization uncertainty of 20 nm and a resolution of 80 nm.

Abbe and Rayleigh defined and formulated the diffraction-limited optical resolution^1^. Several techniques were developed to overcome Abbe’s limit^2^, such as ground-state depletion and single-molecule return (GSDIM^3^, also known as dSTORM^4^), a single molecule super resolution localization microscopy (SMLM) technique based on the *on* and *off* states of fluorescence molecules. Fluorophores bonded to biological structures are embedded in buffers, inducing oxidation and reduction and leading to stochastic blinking. The centers of the point spread functions (PSFs) of single fluorophores are determined in one imaging frame with a sparse number of fluorophores that are emitting photons. By subsequently imaging these emissions, a super resolved pointillistic image is formed^3^. Similar techniques relying on a sparse number of emitting fluorophores per frame, such as photo-activated localization microscopy (PALM, using laser controlled activation of emitting fluorophores) or stochastic optical reconstruction microscopy (STORM), can also be used.

Highly sensitive EMCCD (electron multiplying charge coupled device) imagers were initially used in SMLM instrumentation. Their high sensitivity enhanced the uniformity of the PSFs, increasing the accuracy of PSF centers. Because of limited speed and the presence of excess noise in EMCCDs, a number of researchers started to use faster sCMOS (scientific complementary metal oxide semiconductor) imagers, eventually enabling video rate localization nanoscopy^5^. In this case, faster fluorophores were required^5^,^6^, and faster instrumentation became critical for a dye’s photophysical characterization and optimization^7^,^8^,^9^. To date, the maximal reported frame rate available to SMLM is still limited to 3 000 fps^6^. Single-photon avalanche diode (SPAD) imagers provide even faster frame rates at zero readout noise and could represent an appealing solution for widefield analysis of blinking dyes.

SPAD imagers are arrays of SPADs^10^ that are known for high timing resolution and are thus suitable for time resolved applications such as fluorescence lifetime^11^. These imagers can also be used to count individual photons, so as to form gray scale images at high frame rates and with high photon response uniformity^12^,^13^. When compared to charge accumulating imagers like EMCCD or sCMOS, one of the main advantages of SPAD imagers is an early digitalization of photon counts within the pixel^13^, at the cost of a somewhat lower fill factor – unless recovered by means of microlenses, for instance – and a lower photon detection probability (PDP). A theoretical analysis comparing the three imager types for SMLM applications, including simulations, was presented by Krishnaswami *et al*.^14^.

This article reports on the use of microlens-enabled photon counting SPAD imagers in SMLM. We firstly describe an implementation of a photon counting SPAD imager, called SwissSPAD^12^,^15^ and the optical setup used for the experiments. We then analyze the optimal frame time in SMLM and present the methodology to analyze fluorophore blinking at an unprecedented time resolution of 6.4 μs. Finally, we show the first SMLM images recorded with a SPAD imager.

## SwissSPAD

A SPAD is a pn junction reverse-biased above the breakdown voltage V_BD_ by a voltage known as excess bias. Such a device is said to operate in Geiger mode. The sensitivity of a SPAD, quantified by PDP, and its noise, quantified by dark count rate (DCR), depend on the actual excess bias, albeit in different ways. Since photoresponse in a SPAD is influenced by V_BD_, a uniform breakdown voltage across the die ensures low photoresponse nonuniformity (PRNU), and thus high PDP uniformity. Usually PRNU is very low in SPAD arrays due to low variability of V_BD_ in modern CMOS processes^13^. Because of the early digitalization, the SPAD noise sources are limited to DCR, afterpulsing and crosstalk. SPAD imagers can have very fast frame rates and programmable bit depth resolution without compromising their performance.

**Figure 1:**
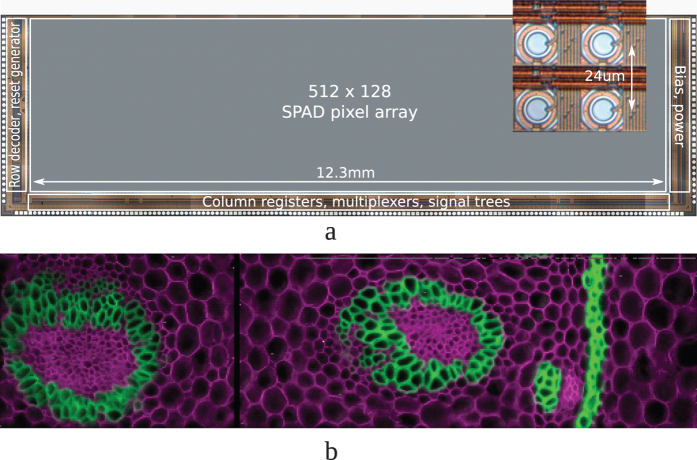
(**a**) Micrograph of the SwissSPAD imager. It has 65 k pixels with 24 μm pixel pitch. It was designed in a 0.35 μm CMOS technology. The peak PDE is about 20% (with microlenses). (**b**) A 512 × 128 SwissSPAD multicolor fluorescence image of a thin slice of a plant root stained with a mixture of fluorescent dyes^35^.

SwissSPAD is an array of 512 × 128 SPAD pixels; Fig. 1a shows a micrograph of the sensor and a detail of the pixels. The pixel comprises a 1-bit memory and a mechanism to quench and recharge the SPAD, thereby achieving global gating over the sensor with deep-subnanosecond accuracy and sub-5 ns width. The 1-bit memory is used to hold the photon hit information, which is subsequently read out using a row select transistor.

Readout is performed using a rolling shutter system with a 6.4 μs frame period. 1-bit frames are sent to a data acquisition board including a field-programmable gate array (FPGA), where multiple frames can be accumulated to form gray level images of programmable bit depth. For an 8-bit image with 255 grayscale values, 255 1-bit frames are needed, leading to a frame rate of 613 fps. For 1 000 fps, 156 1-bit frames are added. When ignoring the nonlinear effect of the imager response^13^, it implies a maximal count of 156 photons per pixel within 1 ms. The 1-bit frame period also determines an upper limit to the effective dead time of the SPAD, since recharge of the 1-bit memory is performed only at the beginning of each frame. If more than one photon is detected by the SPAD within a frame time, it will be interpreted as a single count, introducing a nonlinear response at higher photon rates. To a certain degree, this effect can be corrected for^13^.

In SwissSPAD, the fill factor, i.e. the ratio between the photosensitive area and the overall area of the pixel, is limited to 5%. It can, however, be increased by building a microlens array on the imager to concentrate light on the photosensitive area^16^. By using microlenses, we achieved a concentration factor of 12 for partially collimated light, leading to an effective fill factor of 60% and a peak photon detection efficiency (PDE) of 20% at 450 nm^13^ for an with a f/# of 16. The median DCR is about 200 cps at room temperature with 2% of noisy pixels in the array^12^,^13^.

## GSDIM experimental setup

We used a dual port Leica SR GSD super resolution microscope (Leica Microsystems, Wetzlar, Germany), and initially placed an Andor iXon3 897 BV EMCCD (Andor Technology, Belfast, UK) with a pixel pitch of 16 μm on one port and SwissSPAD with a pixel pitch of 24 μm on the other. We later used a pco.edge 4.2 sCMOS imager (PCO, Kelheim, Germany) in combination with SwissSPAD. The emission light can be directed to either the EMCCD/sCMOS or SwissSPAD. The objective and the tube lens (HCX PL APO 160 × /1.43 Oil CORR GSD) magnifications are 100 × and 1.6 × , respectively. When installing sCMOS, we changed to a 160 × objective with a 1 × tubelens. Yielding a total magnification of 160, the effective EMCCD and SwissSPAD pixel sizes are therefore 100 nm and 150 nm, respectively. When using sCMOS, a demagnificator was used to yield 100 nm pixel size.

For SMLM measurements, we used human bone osteosarcoma epithelial cells (U2OS) stained for microtubuli with Alexa 647, in Vectashield (Vector Laboratories, Burlingame, USA) embedding resin or MEA buffer^17^, as well as fibroblast cells stained for actin with Alexa 647 together with an OxEA buffer^18^, and GATTAquant nanorulers^19^,^20^ (GATTAquant, Braunschweig, Germany).

## Results

We first recorded fluorescence intensity images to compare the imager sensitivity. The ratio between the collected numbers of photons - N_SwissSPAD_/N_EMCCD_ - was found to be 12%^13^. This ratio is lower than the ratio between the peak sensitivities of the two imagers because the emission spectra of dyes are more appropriate for EMCCDs. Figure 1b shows a SwissSPAD multicolor fluorescence image of a Convallaria rhizome (Lily-of-the-Valley) specimen stained with Safranin and Fast Green, having peak excitation wavelengths of 530 nm and 620 nm, respectively. SwissSPAD has an optimum wavelength range from 400 to 600 nm, with a peak at 450 nm, and considerable sensitivity until 750 nm^12^. The EMCCD has its peak between 500 and 700 nm, with a range from 350 to 900 nm. Two consecutive images were taken using different filters and combined to form a single multicolor fluorescence image. It should be noted that Fig. 1b and a 320 × 240^21^ array represent the state-of-the-art in SPAD image quality when compared with images taken with other recent SPAD imagers featuring lower resolution, uniformity and/or fill factor^22^,^23^,^24^. The 320 × 240 SPAD imager features comparable resolution and 26.8% native fill factor, but also asymmetrical pixels and a maximum 1-bit frame rate of kiloframes per second^21^.

### Optimal frame duration

When increasing the speed of the fluorophore blinking to achieve fast SMLM acquisitions, it is not *a priori* clear what frame duration should be used.

**Figure 2:**
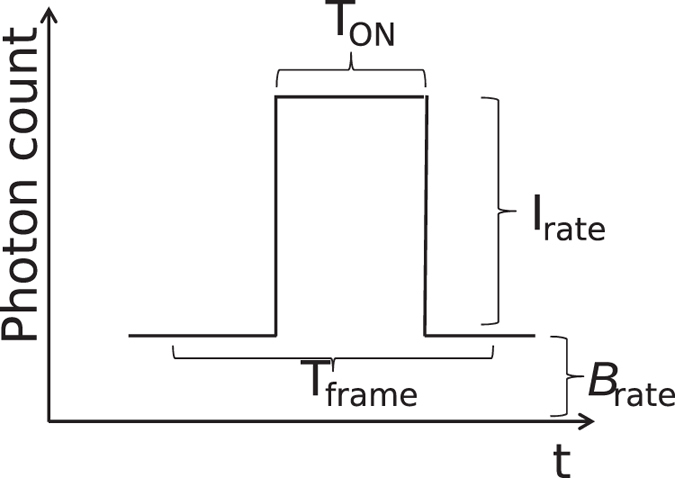
Schematic representation of blinking of a fluorophore in time. *B*_*rate*_ includes the imager noise and sample background noise.

Figure 2 shows a fluorophore photon count rate *I*_*rate*_ in the presence of a background *B*_*rate*_, corresponding to the background accumulated by all pixels, which are contributing to I_*rate*_. Note that more than one pixel contributes to the PSF. The blinking time is assumed to be *T*_*ON*_ and is here assumed to be entirely embedded in a frame. In the general case, the SNR as a function of *T*_*frame*_ is:


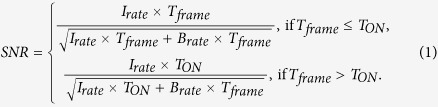


For a constant *T*_*ON*_ value, the maximum SNR is reached for *T*_*frame*_* = T*_*ON*_. This changes when *T*_*ON*_ is an exponential random variable with a decay constant *τ*_*ON*_, which is the case for the emission time of the fluorophores. First, the estimated *E*(*SNR*) with the above assumption for the probability density function of *T*_*ON*_, 
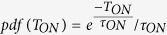
, is calculated as:


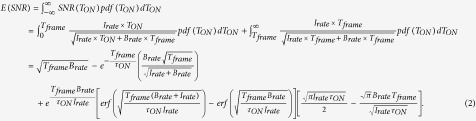


**Figure 3:**
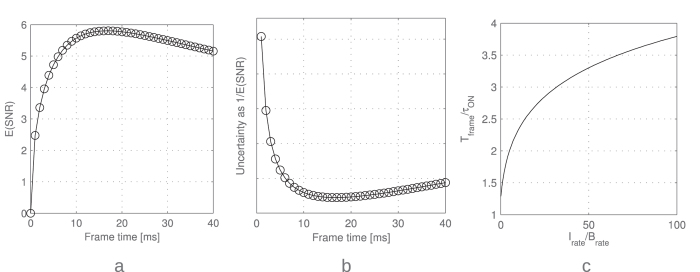
(**a**) SNR of blinking emission as a function of *T*_*frame*_, with *I*_*rate*_/*B*_*rate*_ = 2 and *τ*_*ON*_ = 10 ms. SNR_max_ is reached in this case for *T*_*frame*_ = 1.67 × *τ*_*ON*_. (**b**) The inverse of the SNR is proportional to the expected localization uncertainty. It represents an approximation from the result obtained in ref. 32, where the uncertainty is proportional to the inverse square root of the number of photons in the PSF for the shot noise limited case (*B*_*rate*_ ≪ *I*_*rate*_), and to the inverse of the number of photons for the background noise limited case. (**c**) Optimal *T*_*frame*_/*τ*_*ON*_ ratio as a function of the *I*_*rate*_/*B*_*rate*_ ratio. *T*_*ON*_ is an exponential random variable with a decay constant *τ*_*ON*_. For measurements with lower background, the maximal SNR is reached at longer times, to cover long emissions. For measurements with low fluorophore emission intensities, the maximal SNR is reached close to the average emission duration *τ*_*ON*_.

An example of SNR as a function of *T*_*frame*_ when *T*_*ON*_ is an exponential random variable with a typical decay constant *τ*_*ON*_ = 10 ms is shown in Fig. 3.

The optimal *T*_*frame*_ is found solving:


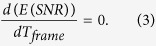


The derivation does not yield an explicit *T*_*frame*_. The optimal *T*_*frame*_ is a function of *I*_*rate*_ and *B*_*rate*_ and can be seen in Fig. 3c. If *B*_*rate*_ = 0, *T*_*frame*_ should be chosen so that *T*_*frame*_ > max(*T*_*ON*_), however, since *T*_*ON*_ is a random variable, the optimal *T*_*frame*_ should be infinite. On the other hand, for *B*_*rate*_ > *I*_*rate*_, *T*_*frame*_ should be chosen so as to minimize the noise effect, thus *T*_*frame*_* = τ*_*ON*_.

**Figure 4:**
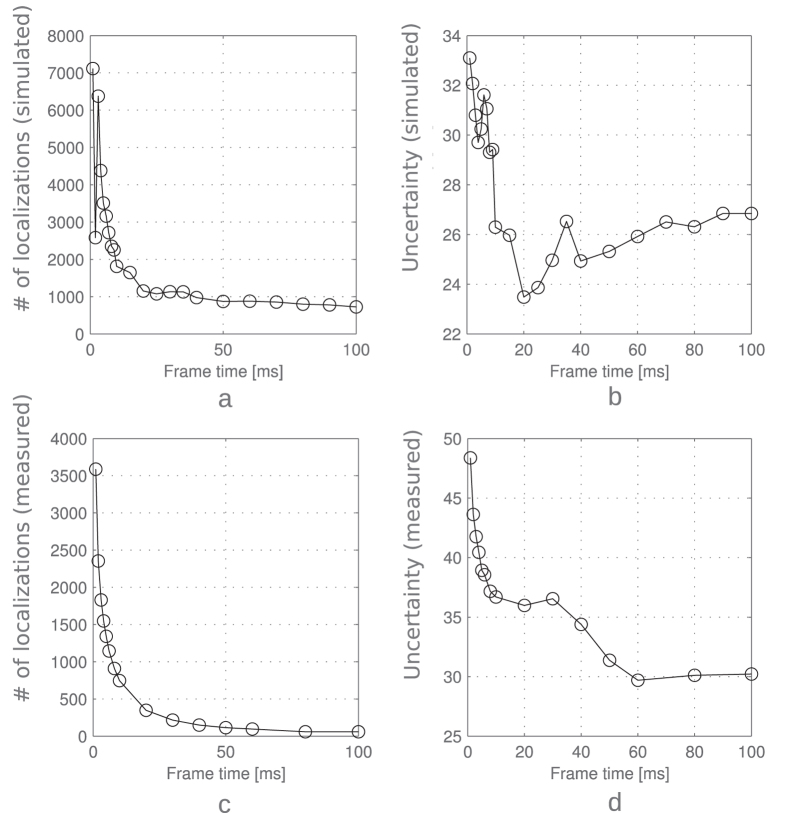
(**a**) Simulated data show how the number of localizations decreases with increasing frame time. With short frame times, the emissions are oversampled and single emitters detected as multiple ones. With longer frame times the emissions start to be rejected. (**b**) The localization uncertainty reaches a minimum at 20 ms. *τ*_*ON*_ was set to be 10 ms, and *I*_*rate*_/*B*_*rate*_ was to 3.6, yielding a theoretical optimum between 18 and 19 ms. The operating point also represents a tradeoff between the number of localizations and the localization uncertainty. (**c**) Measured number of localizations in SwissSPAD over different frame times (1, 2, 3, 4, 5, 6, 8, 10, 20, 30, 40, 50, 60, 80, and 100 ms). (**d**) The localization uncertainty starts saturating after *T*_*frame*_ = 10 ms, but decreases again artificially, most likely due to localization rejections.

SwissSPAD achieves a short frame time without negative effects on the imager performance, such as clock induced charge noise and readout noise. The fast frame time can be used to analyze the optimal *T*_*frame*_. To the best of our knowledge, this analysis has not been carried out before, also due to the fact that the performance of charge accumulating imagers changes as a function of readout speed. Figure 4a and b show ThunderSTORM^25^ super resolution results of simulated data with *τ*_*ON*_ = 10 ms. The background per pixel is constantly increasing when increasing *T*_*frame*_. The fluorophore intensity is also increasing but starts saturating after *T*_*frame*_ > *τ*_*ON*_. The number of localizations decreases rapidly until *T*_*frame*_ = *τ*_*ON*_ (Fig. 4a), while the uncertainty curve reaches its minimum at *T*_*frame*_ > *τ*_*ON*_ (Fig. 4b). Figure 4b is in good agreement with Fig. 3b. Figure 4c and d show experimental SwissSPAD data. The experimental curves resemble the simulated curves, although there is an additional effect of rejecting dim emissions (and partial merging of localizations) causing an artificial uncertainty decrease. Merging close localizations in consecutive frames had insignificant influence on the results presented in Fig. 4, since ThunderSTORM averages the positions, imaged sizes, and backgrounds, and sums the intensity. Adding photons from multiple frames and then calculating the position and uncertainty could however yield a decrease in uncertainty^26^, but it requires intense processing, currently not implemented in super resolution software.

**Figure 5:**
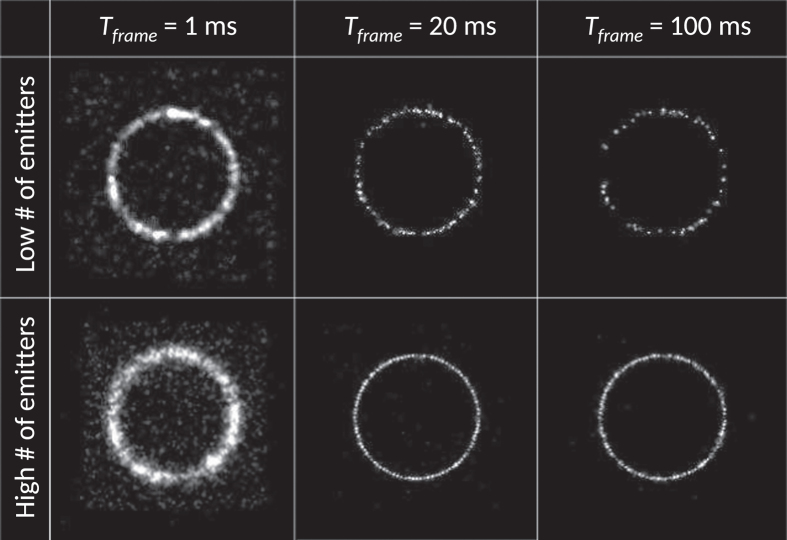
Simulated super resolution images of molecules randomly blinking at positions in a circle, obtained with different frame times. *τ*_*ON*_ = 10 ms. If the blinking is oversampled, the uncertainty is high. If the blinking is undersampled (100 ms), the image will both yield a lower number of localizations (Low # of emitters) and higher localization uncertainty (High # of emitters). A *T*_*frame*_ of 20 ms represents the optimum. *I*_*rate*_/*B*_*rate*_ was set to be 3.6, yielding a theoretical optimum between 18 and 19 ms.

Figure 5 shows simulated SMLM results on emissions in a circle using three different *T*_*frame*_, where the theoretical optimum is between 18 and 19 ms. Short frame times result in larger number of localizations (also faulty ones) with high localization uncertainty, while long frame times result in localization rejection and worse localization uncertainty.

### Blinking characterization

**Figure 6:**
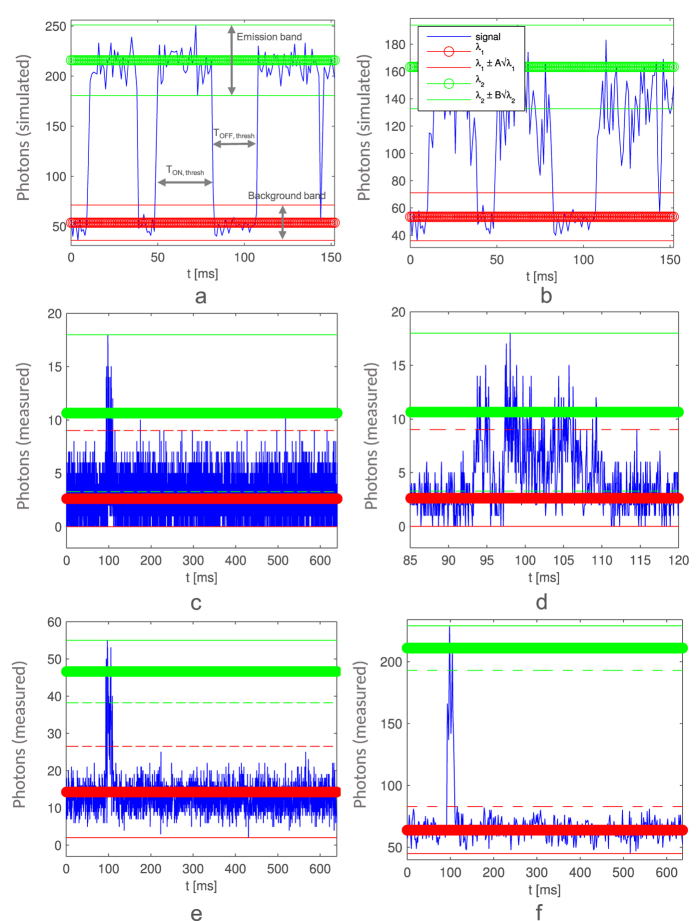
Stochastic blinking, where the molecule switches between an *on* and an *off* state: simulated photon response without fast blinking (**a**); simulated photon response with fast blinking, where the signal exhibits many outliers situated outside the estimated Poisson band (**b**). SwissSPAD-measured molecule blinking with 0.064 ms (**c**,**d**), 0.3 ms (**e**), and 1.6 ms (**f**) accumulation time. In particular (**d**) shows the zoomed blinking, at around *t* = 100 ms, with 0.064 ms accumulation time. Note that the emission and background bands overlap in (**c**,**d**). We used a U2OS sample with MEA buffer. Also note that if additional fast blinking is present, the photon response may be larger than expected from Poisson statistics alone. The upper and lower estimated boundaries for the photon response are marked with thin green (emission) and red (background and noise) lines, while the estimated averages are shown with thicker lines.

When using higher power intensities to reach fast emission times, one of the most important procedures is to characterize the dye’s blinking to set the optimal operating point. An example of blinking is shown in Fig. 6a. *On* states that appear continuous could additionally exhibit fast blinking (Fig. 6b) not clearly observable with 1–10 ms resolution time frame. This fast blinking can however be detected when using Poisson statistics. To estimate the *on* and *off* averages as well as the corresponding emission and background bands (see Fig. 6a), we first used the whole signal sample length to estimate *A*, where:


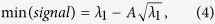


and thus *λ*_1_, i.e. the average value of the background noise as random variable with Poisson distribution, where:


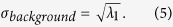


Note that the whole signal includes the background and the emission signal, and that min(*signal*) and max(*signal*) are easily found. The emission sample length, i.e. the total length of the signal above max(*background*), is then used to find *B*, where:


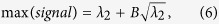


and thus *λ*_2_, i.e. the average value of the emission signal and background noise as random variable with Poisson distribution.

In the presence of additional fast blinking (due to a triplet state or additional dark states), the photon response will indeed not follow Poisson statistics with a constant emission rate, as it is clear from Fig. 6b. The emission changes in time fall clearly outside the estimated Poisson band.

To analyze such a fast blinking phenomena, shorter accumulation times must be used. However, due to the decrease of accumulation time, the SNR is reduced as well, leading to overlapping of the signal and background bands. We therefore used two methods for molecule blinking analysis: a thresholding method, which is suitable for signals with two distinguishable signal intensities (molecule *on* and molecule *off*), where the bands are well separated, and an autocorrelation method, which can be used for continuous signals, in cases where the SNR is not high enough to distinguish clearly between the *on* and *off* states^27^.

After the ThunderSTORM^25^ analysis, we extracted the single molecule photon response for the U2OS sample with MEA buffer. We used 5 × 5 pixels to cover the whole molecule emission PSF. Examples of measured blinking with different accumulation times, with estimated emission and background bands, are shown in Fig. 6c–f.

We expect the emissions to have two blinking components: slow and fast. The number of emitting fluorophores per frame is sparse due to the slow component. The fast component is not an application necessity, and is induced by the triplet state or other dark states. *τ*_*OFF*_ is defined as the time between two emissions of the same molecule. We recorded images with three different laser intensities and firstly used the thresholding method to extract *τ*_*ON*_ and *τ*_*OFF*_ of the slow blinking component. Fitted monoexponential distributions for the *on* time have *τ*_*ON*,*thresh*_ = 6.1 ms, *τ*_*ON*,*thresh*_ = 3.1 ms and *τ*_*ON*,*thresh*_ = 2.0 ms for laser powers of 5.7 kW/cm^2^, 8.5 kW/cm^2^ and 11.3 kW/cm^2^ respectively. *τ*_*ON*,*thresh*_ decreases with increase of laser intensities. Although *off* times over a wide range of laser intensities should be analyzed as a sum of three exponential distributions^6^, we fitted *off* data monoexponentially as an indication of changes over different laser intensities. The distributions for the *off* time have *τ*_*OFF*,*thresh*_ = 13.4 ms, *τ*_*OFF*,*thresh*_ = 5.7 ms and *τ*_*OFF*,*thresh*_ = 3.3 ms for laser powers of 5.7 kW/cm^2^, 8.5 kW/cm^2^ and 11.3 kW/cm^2^ respectively. The threshold was set at the upper noise bound because of possible multiple emissions with different photon intensities and additional fast blinking (see Fig. 6). Single outliers over the upper noise bound were rejected. A fast sCMOS camera can extract *τ*_*ON*,*thresh*_ and *τ*_*OFF*,*thresh*_ in the range of 1–10 ms, but cannot investigate the additional fast blinking in the μs range, whereas a SPAD imager such as SwissSPAD can.

The additional fast blinking can lower the overall molecule emission. When compared to our previous work^28^ where we used the thresholding to estimate the beginning and end of course emission, here we used the information of the beginning and end of the molecule emission from 8-bit video ThunderSTORM analysis. The 8-bit data was formed by accumulating the original 1-bit data in the PC. We than returned to the 1-bit data with 6.4 μs time resolution and used autocorrelation to explore if additional fast blinking is present. Note that no information was lost in the process. Since we expect the blinking to be exponentially distributed, the autocorrelation curve is fitted to extract a decay constant of the exponential autocorrelation curve *τ*_*auto*_. The drawback of using 1-bit data is that the emission and noise bands (as shown in Fig. 6) are not easily distinguishable and *τ*_*auto*_ will be a combination of the fast *on* and *off* lifetimes (if present), i.e.:


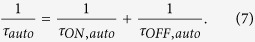


**Figure 7:**
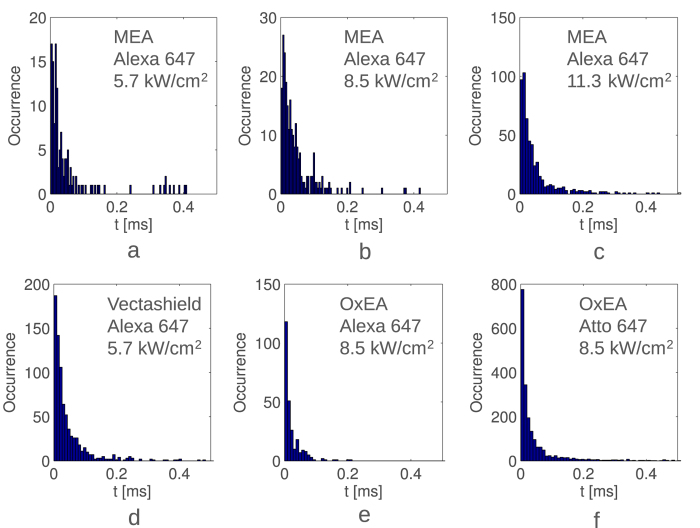
SwissSPAD measured autocorrelation decay distributions for different laser intensities of 5.7 kW/cm^2^ (**a**), 8.5 kW/cm^2^ (**b**) and 11.3 kW/cm^2^ (**c**), of Alexa 647 with MEA buffer. (**d**) Shows the distribution of Alexa 647 with Vectashield. Plots (**e**,**f**) show the distributions of Alexa 647 and Atto 647 with OxEA buffer, respectively.

**Figure 8:**
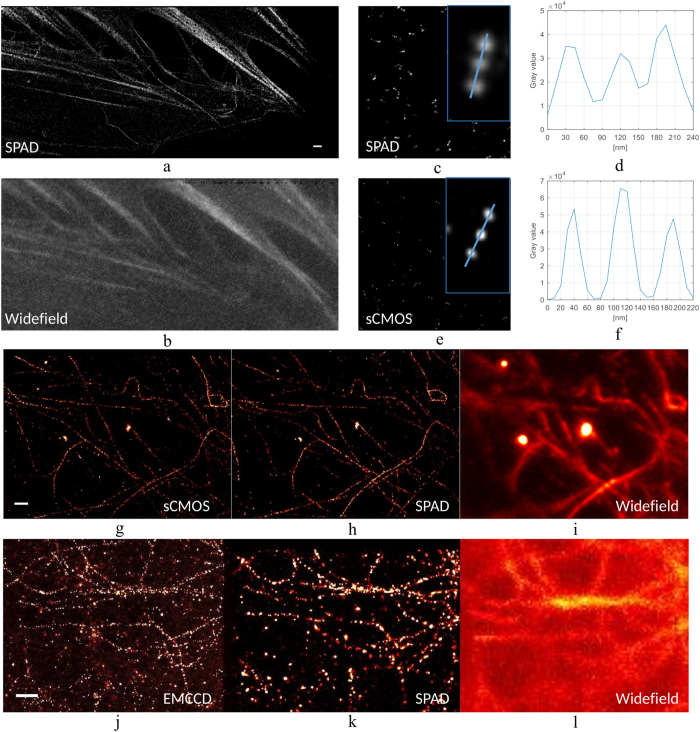
(**a**) SwissSPAD super resolution image of actin labeled with Alexa 647 in an OxEA buffer compared to (**b**) the widefield image. The white bar shows 1 μm. (**c**,**d**) Shows SwissSPAD GATTAquant PAINT 80 R nanorulers where emitter are separated by 80 nm. (**e**,**f**) Shows the nanoruler imaged with sCMOS. (**g**) sCMOS and (**h**) SwissSPAD super resolution images of microtubuli compared with (**i**) a widefield image taken with a sCMOS. The white bar shows 1 μm. (**j**–**l**) Show an U2OS cell stained with Alexa Fluor 647, in Vectashield.

In our U2OS sample with microtubuli stained with Alexa 647 and with MEA buffer, we measured 68%, 74% and 70% emissions with 0 < *τ*_*auto*_ < 1 ms. The rest of the blinks feature a *τ*_*auto*_ = −∞, indicating no blinking (autocorrelation is flat), and a minor number of outlying *τ*_*auto*_ because of the low number of emission photons. The averages *τ*_*auto*_ of this fast blinking were 58 μs, 48 μs and 54 μs for laser intensities of 5.7 kW/cm^2^, 8.5 kW/cm^2^ and 11.3 kW/cm^2^ respectively (Fig. 7a,b and c). The U2OS sample stained with Alexa 647, but with a Vectashield embedding resin, had 46%/53 μs (Fig. 7d), 55%/58 μs and 41%/57 μs for laser intensities of 5.7 kW/cm^2^, 8.5 kW/cm^2^and 11.3 kW/cm^2^ respectively. A Fibroblast sample stained with Alexa 647 in an OxEA buffer^18^ had an average *τ*_*auto*_ of 28 μs, where 20% emissions had 0 < *τ*_*auto*_ < 1 ms (Fig. 7e). When using Atto 647 in an OxEA buffer, we detected 27% emissions with 0 < *τ*_*auto*_ < 1 ms, with an average of 49 μs (Fig. 8f).

Data shows that laser power has a small effect on the fast blinking. On the contrary, buffers change blinking parameters significantly, indicating that the fast blinking is caused by the triplet state. The two different dyes also have a different blinking behavior. The extracted *τ*_*auto*_ (Fig. 7) - with an unprecedented time resolution range down to 6.4 μs - can be used for the photophysical analysis of a dye and its optimization. Timing parameters could also be used to estimate pH or concentration values^7^,^29^.

### Super resolution images

We compared SwissSPAD, sCMOS and EMCCD super resolution measurements. When comparing SwissSPAD and EMCCD/sCMOS we alternately recorded frames with the two imagers. To compare SwissSPAD and sCMOS, we also used GATTAquant PAINT 80 R nanorulers, where three emitters in each nanoruler are separated by 80 nm^20^. The SwissSPAD has a PDE of around 9% at the emission wavelength of Alexa Fluor 647 and Atto 655.

The SwissSPAD video was first pre-processed in MATLAB to correct for the sensor’s nonlinear photon response and DCR, on a pixel-by-pixel basis, employing the following correction scheme^13^:


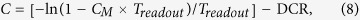


where *C* represents the corrected count rate, *C*_*M*_ the measured count rate, and *T*_*readout*_ = 6.4 μs the dead time of the pixel.

The recorded 1-bit, 6.4 μs frames were then binned to form 10 ms frames, background was subtracted^30^ and images were analyzed with ThunderSTORM^25^. Figure 8a shows a super resolved image of actin in a fibroblast sample, stained with Alexa Fluor 647, used with an OxEA buffer. Figure 8b shows the corresponding widefield image. Figure 8c–f show GATTAquant PAINT 80 R nanoruler data of SwissSPAD and sCMOS, respectively. Images where reconstructed using 5 000 10 ms frames. When zoomed, one can note the resolvability of 80 nm distances between single emitters, where sCMOS has a 2–3 times finer resolution. Gyongy *et al*. showed the benefits of aggregating short frames with a single GATTAquant nanoruler imaged with a SPAD imager^26^,^31^, but without super resolution images.

Figure 8g–i show microtubuli from a fibroblast sample, which was stained with Alexa Fluor 647, and prepared in OxEA buffer. The sCMOS and SwissSPAD images contain 40 000 localizations. Although the effect is minimized by preprocessing (see Equation 8), some of the noisy pixels distort localizations. Highly noisy pixels, known as screamers, constitute 2% of the overall pixel population^13^; interpolation is used to minimize localization distortions. The interpolation of hot pixels caused loss of data and a localization uncertainty increase. This effect was verified by the software, resulting in a bias of the localization position (away from the true position).

Figure 8j–l show a SwissSPAD super resolution image of microtubuli in an U2OS cell stained with Alexa Fluor 647, in Vectashield. It was obtained during a 70 second exposure and contains 90 000 localizations.

Thanks to the implementation of microlenses that boosted the SwissSPAD PDE, we obtained the first SMLM images recorded with a SPAD imager. The sCMOS typically collected 800 photons with a localization uncertainty of 10 nm (for Fig. 8g), while SwissSPAD had 100 photons collected with 20 nm uncertainty (for Fig. 8h). For the measurements with EMCCD, the typical estimated number of collected photons was 1800, and the typical estimated localization uncertainty about 15 nm (Fig. 8j), while SwissSPAD had 200 photons collected and 30 nm uncertainty (Fig. 8k). The emphasis should be placed on the comparison between the SPAD and one of the two other imagers. A direct comparison between the sCMOS and EMCCD images and uncertainty results is not completely fair since the sCMOS imaged samples with OxEA, and the EMCDD imaged samples with Vectashield. The localization uncertainty is estimated using ThunderSTORM, employing the Thompson *et al*. formula^32^ for sCMOS and SPAD, and Quan *et al*. formula^33^ for EMCCD (as to include the multiplication noise). It is worth mentioning that, although the EMCCD collected 10 times more photons, the localization uncertainty is only about two times better. Ten times more photons should yield √10 = 3.16 better localization uncertainty, but the excess noise lowers this by a factor of √2^6^, resulting in √5 = 2.23 better localization uncertainty. CMOS SPAD structures reaching a PDP of 40% between 440 and 620 nm have been published^34^, but not yet implemented as SPAD imagers. A theoretical analysis did actually show that SPAD imagers with the same sensitivity as EMCCD and sCMOS imagers will feature superior localization accuracy because of absence of excess and readout noise^14^, assuming that the dark noise uniformity is similar to conventional CMOS imagers.

## Conclusions

SPAD imagers have by some been regarded as unsuitable for applications where sensitivity is critical. This belief was driven by the lack of high fill factor sensors. With the introduction of SPAD imagers with improved fill factor, we could demonstrate that super resolution localization microscopy can exploit the high timing resolution provided by this type of imagers. We showed the first super resolution localization microscopy results obtained with a SPAD imager, with an estimated localization uncertainty of 20 nm and resolution better than 80 nm. We investigated the optimal frame time and concluded that it is longer than the average blinking time, and dependent on the emission intensity and background intensity ratio.

We also presented the instrumentation and methodology for a systematic widefield blinking analysis. We believe that the characterization of very fast blinking is critical for future developments in super resolution, with the goal of high acquisition speeds for the best possible localization uncertainty. Finally, we presented data where the MEA buffer allowed for 70% of emission with additional fast blinking in the μs range, whereas OxEA allowed for 20%. To the best of our knowledge, this is the first comprehensive widefield analysis of blinking with microsecond timing resolution and the first performed on a SPAD imager.

State-of-the-art SPAD imagers have a competitive advantage when combining spatial and temporal resolution, but still don’t reach the same spatial resolution as EMCCD and sCMOS due to the lower PDE. Further developments of the SPAD imagers will yield a higher PDE and a smaller pixel pitch, which is expected to further increase the noise uniformity and lower the number of hot pixels.

## Additional Information

**How to cite this article:** Antolovic, I. M. *et al*. SPAD imagers for super resolution localization microscopy enable analysis of fast fluorophore blinking. *Sci. Rep.* **7**, 44108; doi: 10.1038/srep44108 (2017).

**Publisher's note:** Springer Nature remains neutral with regard to jurisdictional claims in published maps and institutional affiliations.
